# A 3D genome compendium of breast cancer progression

**DOI:** 10.1016/j.isci.2025.113268

**Published:** 2025-08-05

**Authors:** Teun van den Brand, Maria Donaldson Collier, Koen D. Flach, Sebastian Gregoricchio, Isabel Mayayo-Peralta, Zhanna Dauyey, Karianne Schuurman, Hans Teunissen, Wilbert Zwart, Elzo de Wit

**Affiliations:** 1 Division of Gene Regulation, Netherlands Cancer Institute, Amsterdam, the Netherlands; 2 Division of Oncogenomics, Oncode Institute & Netherlands Cancer Institute, Amsterdam, the Netherlands

**Keywords:** Genomics, Chromosome organization, Molecular Genetics, Cancer

## Abstract

During cancer development and progression massive alterations in gene expression are observed. Gene regulation occurs within the context of the 3D genome. However, the impact of disease progression on 3D genome organization remains poorly understood. Using breast cancer as a model, we have profiled the 3D genome throughout the natural course of the disease; from development to progression. Uniquely, we analyzed tumors from the same patients, enabling us to gauge the extent of changes that happen upon metastasis. Our results show that the organization of the genome at the level of topologically associating domains (TADs) and compartments upon tumorigenesis and metastasis, is remarkably stable. However, in pleural metastases, representing heavily pretreated progressive disease, the 3D genome is massively affected, and highly heterogeneous between patients, both on the compartment and TAD level. Our data reveal that disease progression in breast cancer is associated with a progressive unraveling of the 3D genome.

## Highlights

• We have generated Hi-C maps in paired primary and metastatic breast cancer samples
• Changes in the 3D genome between primary and metastatic lesions are limited
• Pleural effusion samples show massive heterogeneity at TAD and compartment level
• ERα is associated with multi-megabase chromatin interactions in breast cancer

## Graphical abstract

**Figure:**
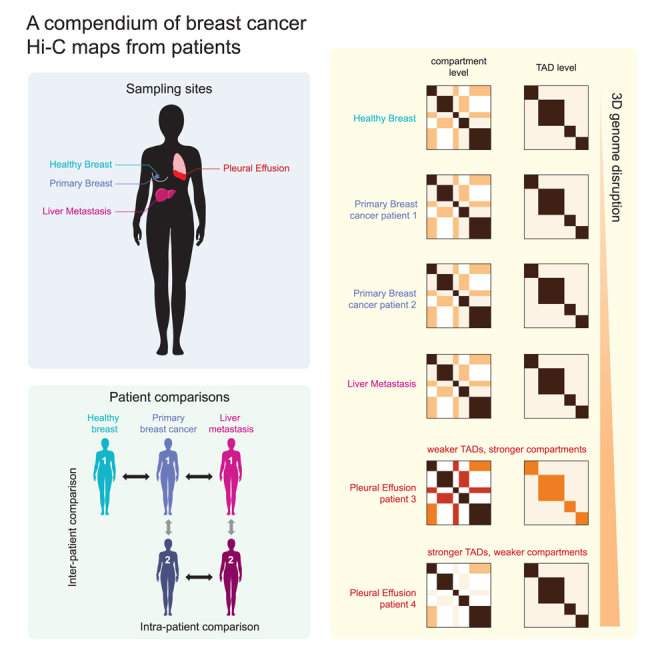

## Introduction

The human genome measures ∼2 m in length, but fits into a nucleus that is between 5 and 10 μm in diameter. To facilitate essential nuclear functions, such as DNA replication, DNA repair, and gene expression, the genome is folded in a non-random manner.^1^ Proximity ligation methods such as Hi-C enable the genome-wide measurement of bulk organization of the 3D genome.^2^^,^^3^ Previous experiments in healthy tissues and cell lines have provided a comprehensive view of the 3D genome at multiple scales.

Hi-C experiments have confirmed microscopy experiments that showed that chromosomes occupy their own nuclear subspace, termed chromosome territories.^4^^,^^5^ Within these territories, active and inactive chromatin is segregated into A and B compartments, respectively.^3^ A compartments are generally gene rich, active and in the nuclear interior, whereas B compartments tend to be relatively gene poor, transcriptionally silent and more at the nuclear periphery. These compartment domains can be multiple megabases in size, although the size of the identified domains can also depend on the resolution of the Hi-C data. This so-called compartmentalization, is manifested as a “plaid” or “checkerboard” pattern in Hi-C data. Topologically associating domains (TADs) represent another layer of 3D genome organization.^6^^,^^7^ TADs appear in the Hi-C matrix as squares or triangles 100 kb–2 Mb in size. They are formed as a consequence of cohesin-mediated loop extrusion,^8^ in which small chromatin loops are progressively increased in size in an ATP-dependent manner.^9^^,^^10^ Loop extrusion is halted by the insulator protein CTCF bound to DNA.^11^^,^^12^^,^^13^ TADs are dynamic structures representing an average state of the 3D genome.^14^^,^^15^ Loop extrusion can also bring together two distal CTCF sites into CTCF-anchored chromatin loops, which can be seen as dots in a Hi-C matrix.^16^

In this study we chart the changes in the 3D genome during cancer development and progression. Cancer arises from a complex interplay between genetic and epigenetic alterations, leading to dysregulation of cellular control mechanisms.^17^ This includes mutations in tumor suppressor genes or proto-oncogenes, resulting in uncontrolled proliferation, impaired cell death, and ultimately tumor formation. Metastasis—the process of cancer cells migrating from the primary tumor to distant sites—is also coupled with significant changes in gene expression.^18^^,^^19^

The 3D genome has been extensively characterized in human cancer cell lines.^16^^,^^20^^,^^21^^,^^22^^,^^23^ Direct comparison of the breast cancer cell line MCF-7 and the epithelial mammary cell line MCF-10A revealed that 12% of compartments were switched and that this correlated with difference in gene expression.^24^ Furthermore, TAD boundaries were largely consistent between the two cell types. Stimulation of the breast cancer cell line T47D with a progesterone analogue revealed that ∼80% of TAD borders remained the same after treatment.^25^ More recently, Hi-C experiments have charted inter-patient heterogeneity in acute lymphoblastic leukemia.^26^^,^^27^ Given the amount of material required for Hi-C experiments, blood cancers were the first cancer types to be profiled. More recently, the first studies employing Hi-C technologies on solid tumors^21^ including colorectal cancer,^28^^,^^29^ prostate cancer,^30^ breast cancer,^31^ and glioblastoma,^32^ have been reported. Although these studies have been highly insightful with respect to our understanding of the 3D genome in cancer, how the 3D genome changes throughout the natural course of cancer development and progression—from healthy tissue to primary cancer and eventually lethal metastatic disease in the same patient—is currently unknown.

For our analyses we used breast cancer as a model. Breast cancer is the most-common diagnosed cancer in women, with over 2.3 million women new diagnoses each year, and over 500,000 patients succumb to the disease.^33^ With this, breast cancer is the second-most common cause of cancer-related death.

Around 80% of all breast cancers express the estrogen receptor alpha (ERα), and are considered to be dependent on the transcriptional activity of this hormone-mediated transcription factor for their growth.^34^ ERα mainly acts through *cis*-regulatory elements, which communicate with promoter regions to drive transcriptional programs that govern tumor cell proliferation,^35^ within the context of the 3D organization of the genome.^36^ Due to the direct causal impact of ERα action on luminal cancer growth, endocrine therapy is the standard-of-care for this patient population, but resistance to treatment is commonly observed.^37^

In this study, we aimed to understand how the 3D genome is altered during breast cancer development and progression, *in situ*. Toward this goal, we collected cells from patient biopsies and performed Hi-C on four types of biopsies representing different stages of disease progression: healthy mammary tissue, primary breast (PB) tumors, liver metastasis, and malignant pleural effusions. We survey the changes in the 3D genomes at the level of structural variation (SV), compartments and TADs, as well as ERα associated distal interactions. The major pattern we uncover is the gradual yet progressive increase in 3D genome heterogeneity during tumor progression.

## Results

### A Hi-C dataset charting tumor progression

**Figure 1:**
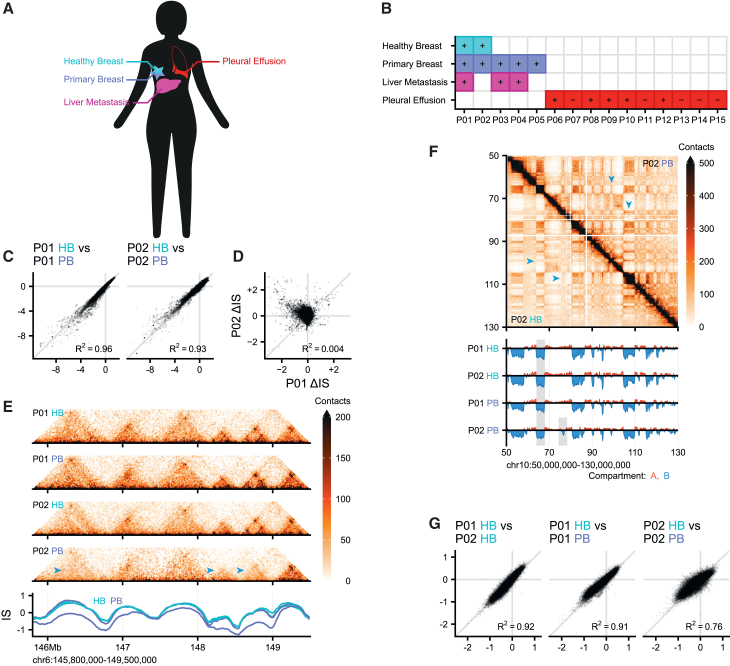
The 3D genome of primary breast cancer samples are not dissimilar to healthy breast samples (A) Schematic representation of samples included in this study. (B) Overview of collected samples, showing for which patients (*x* axis) what biopsies (*y* axis) were collected. Estrogen receptor alpha (ERα) status is indicated with “+” for positive and “–” for negative. (C) Correlation plots for genome-wide insulation scores (IS) in comparing P01 and P02. (D) Differences in IS between HB and PB comparing P01 and P02. (E) Example TAD structures in a healthy breast (HB) and primary breast (PB) sample of the same patient. Insulation scores below are for P01 and P02. A region that is more insulated in PB than in HB is indicated in gray. (F) Example compartmentalization in HB and PB sample of the same patient. Compartment score below indicates a weakened B-compartment in PB samples, and a P02 PB specific compartment switch (gray areas). Blue arrows indicate differences between samples. (G) Correlation plots for genome-wide compartment scores in selected comparisons.

To better understand how the 3D genome is affected in breast cancer progression, we selected biopsies from donors at various stages of breast cancer disease progression and performed *in situ* Hi-C. Biopsies were collected from matched healthy breast (HB) tissue (*n* = 2), PB cancer (*n* = 4), and liver metastases (LM) (*n* = 4) (Figures 1A and 1B). All matched tumor samples collected (P1-4) were diagnosed as ERα-positive luminal subtype tumors and received adjuvant tamoxifen to block ERα (Figure 1B). All these patients developed resistance within five years of starting treatment with tamoxifen and received another endocrine therapy (fulvestrant or aromatase inhibitors). Of note, all these patients additionally received surgery as well as chemotherapy and/or radiation, but eventually relapsed and developed metastasis to the liver, where diagnostic biopsies were collected. All solid tumor samples analyzed in this study have high tumor cell percentage (≥70%) and retained high ERα expression in greater than 80% of the tumor, despite prolonged endocrine therapy, as confirmed by IHC staining (data not shown).

From an independent series, malignant pleural effusions (PE) from metastatic breast cancer patients were analyzed as well (*n* = 10), representing a very advanced disease state, in which both luminal ERα-positive (*n* = 7) and basal-like ERα-negative (*n* = 3) tumors were selected (Figures 1A and 1B). Patient characteristics and clinical parameters are summarized in Table S1. Jointly, this collection tumor samples provides a unique opportunity to analyze changes in the 3D genome in the development and progression of human breast cancer in a clinically highly relevant context.

### Tumorigenesis has limited impact on 3D genome organization

To understand how 3D genome regulation is altered in tumorigenesis, we first sought to understand differences between HB tissues and primary tumors. For this comparison, we can take advantage of the paired nature of healthy and primary biopsies in two patients: P01 and P02.

TADs are thought to mediate gene regulatory interactions. The position and strength of TAD boundaries is typically measured using the “insulation score”.^38^ We calculated the genome-wide insulation score and noted, to our surprise, that it was highly comparable between the HB and the primary tumor. Insulation correlated well between healthy tissue and primary tumors (R^2^ ≥ 0.93), suggesting that changes are relatively mild (Figure 1C). To understand whether there are any consistent changes in TAD insulation, we calculated the differential insulation score (ΔIS) between the healthy tissue and primary tumor, for both patients. The ΔIS correlated poorly between P01 and P02 (R^2^ < 0.01), indicating very few consistent changes in insulation by progressing from healthy tissue to primary tumor (Figure 1D). Despite the high similarity in insulation score between tissue states, we did note several patient-specific local insulation changes in the Hi-C maps, when comparing healthy tissue and primary tumor. Figure 1E shows an example of a difference in TAD strength on chromosome 6 in P02 between HB and primary tumor (indicated by the blue arrows), whereas the Hi-C signal in patient P01 is very comparable. Our analyses of the insulation scores suggest that tumorigenesis has limited effect at the TAD level and, if changes occur, they are patient-specific.

Active and inactive chromatin is segregated in the nucleus in a process termed compartmentalization. Compartment identity has been shown to differ substantially between different tissue types^20^^,^^39^ correlating with gene expression status in the compartment domains. Figure 1F shows a Hi-C heatmap for the p-arm of chromosome 10 comparing the compartment organization of the HB and primary tumor for patient P02. This plot shows the archetypical plaid pattern associated with compartmentalization.^3^ Genome-wide compartmentalization can be extracted to a one-dimensional score called the “compartment score” where a positive score indicates the A (active) compartment and a negative score indicates the B (inactive) compartment. At the position of ∼68 Mb, a mild weakening of a strong B compartment block is indicated that is consistent over tumorigenesis, i.e., the progression from HB to PB, for both patients. In patient P02 specifically, an A-to-B compartment switch appears at ∼75 Mb that leads to increased interactions with other B-compartments in the primary tumor (Figure 1F, arrows). However, no considerable A-to-B compartment switches are observed for patient P01 on this chromosome arm. In fact, when the genome-wide compartment score is compared for P01, they are highly similar and the variation is relatively minor (Figure 1G, R^2^ > 0.9). For P02, the compartment score comparison between HB and primary tumor is also quite similar, although the variation is more extensive (Figure 1G, R^2^ = 0.75). In conclusion, concerted compartment level changes between healthy tissue and primary tumor are relatively rare. Furthermore, observed differences are not consistent between patients.

At the transition from healthy tissue to PB cancer, we conclude that differences within patients can be observed, but that consistent changes upon tumorigenesis tend to be limited.

### 3D genome changes associated with disease progression

Our unique dataset of paired primary and metastatic samples allows us to explore the differences in 3D genome organization upon disease progression.

**Figure 2:**
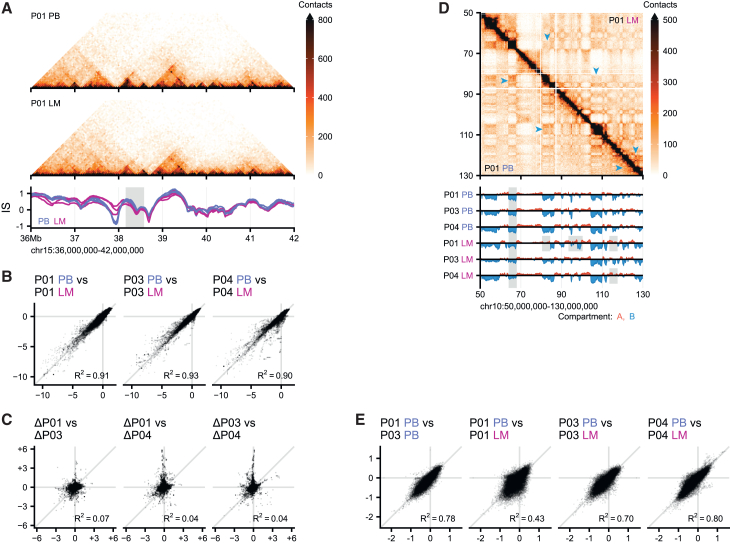
Between primary tumors and metastases, the 3D genome has adapted mostly by means of compartmentalization (A) Example TAD structures in a primary tumor (PB) and liver metastasis (LM) in P01. Insulation scores below are for patients P01, P03, and P04. A region that loses insulation in liver metastases is indicated in gray. (B) Correlation plots for genome-wide insulation within patients. (C) Correlation of difference in insulation between patients. (D) Example compartmentalization in PB and LM sample of the same patient. Compartment scores below weakening of a B compartment and various patient-specific changes (gray areas). Blue arrows indicate differences between samples. (E) Correlation plots for genome-wide compartment scores in selected comparisons.

We first tested whether there were consistent changes at the level of TAD organization. We found that there are regions in the genome with consistent changes in the insulation score between primary tumor samples and metastatic samples. When we use a threshold for the insulation score difference of 0.25 and at least 10 consecutive bins that are different, we find 188 regions with altered insulation score upon tumor progression to metastatic disease, corresponding to ∼66 Mb of the genome. Note that a substantial fraction of these sites are located close to the centromere or telomere which might be indicative of technical variation in the data. One example is on chromosome 15, where a strong TAD boundary around 38 Mb becomes weaker in all metastatic samples (Figure 2A). Despite these consistent changes, globally, insulation scores between paired primary and metastatic samples correlate well (R^2^ > 0.9, Figure 2B), suggesting that the changes in the 3D genome are mild. Furthermore, when we compared the differences in insulation scores, we found that they are uncorrelated between patients (R^2^ < 0.1, Figure 2C), suggesting that most changes at the TAD level are patient-specific. Unfortunately, the depth of our Hi-C maps precludes reliable calling of loops at the level of the individual samples. However, by combining all the HB, primary tumor and metastatic samples we were able to perform compound loop calling using ChromoSight.^40^ This resulted in the identification of 15394 loops. Next we used aggregate peak analysis (APA) to determine whether there were substantial differences in the loop strength between samples or tumor stages (Figure S1). Our analyses did not point toward large changes in loop strength. These results are consistent with the lack of differences between samples for the TAD boundary insulation.

Next, we considered whether the transition from the primary tumor to metastatic disease affects compartmentalization. We focused on the p-arm of chromosome 10, where we found substantial changes in compartment strength and identity for P01 (Figure 2D). Interestingly, these changes seem to be specific to P01 as we find only one region on this chromosome arm that shows a consistent change between the primary tumor and the metastatic sample. Coincidentally, this is the same region that shows an increase in compartment score between HB and primary tumor for the same patient. To determine the systematic changes in compartmentalization upon tumor progression, we compared the genome-wide compartment scores for primary tumor and metastatic samples. The changes in the compartment score that we observed for P01 are a genome-wide phenomenon as evidenced by the low correlation (Figure 2E, R^2^ = 0.43). However, for P03 and P04, the correlations between the compartment scores of primary and metastatic samples are much higher (R^2^ = 0.70 and R^2^ = 0.80, respectively). Although the correlation in compartment score is lower than we observe between two HB tissue samples (Figure 1C), it is important to indicate that large switches in the compartment score are notably absent in two out of three samples. These results indicate that, while compartment changes can occur during disease progression, compartment identity is frequently maintained during metastasis to a distal site.

### Cataloging structural variation in breast cancer using Hi-C

Cancer is associated with chromosome instability (CIN), which results in copy number variation (CNV) and SV. Because Hi-C is a genome-wide assay, it allows us to infer CNVs and because it captures long-range chromatin interactions, it enables the identification of copy number neutral translocations without the need for high coverage at the breakpoint locations.

**Figure 3:**
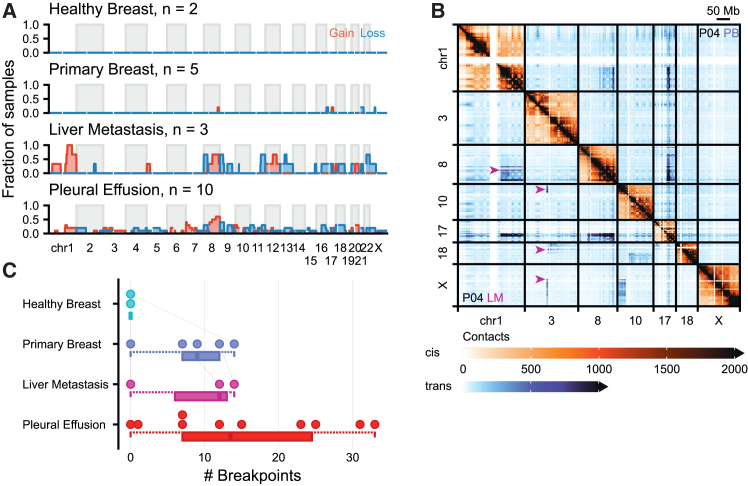
Structural instability accumulates as breast cancer progresses (A) Summary of copy number variations found in each biopsy type across genomic locations. A region was considered a gain when the log2 ratio exceeded log2 1.25 and considered a loss when that ratio succeeded log2 0.75. (B) Example Hi-C map of multiple chromosomes showing an increase in intrachromosomal rearrangements in P04 progressing from primary breast (PB) to liver metastasis (LM). Arrows indicate rearrangements found in LM but not in PB. (C) Summary of intrachromosomal rearrangements across biopsy types, where a rearrangement is counted by detected breakpoints. Breakpoints were detected by manual classification of Hi-C contact maps blinded for sample identity in randomized order. Lines between points indicate related samples from the same patient. Box-and-whiskers plot indicate data distribution: boxes indicate the interquartile range (25–75%), the whiskers extend to 1.5 time the interquartile range from the edge of the box or to the smallest or largest value.s.

For the purpose of estimating CNVs, read pairs were decoupled and mate pairs were counted individually in bins across the genome. After correcting for GC-bias, mappability, and the number of restriction sites, we used the DNACopy software^41^ to infer copy numbers. Because tumors can be heterogeneous in their composition^42^ and—given the high tumor cell percentage of our samples—a minor fraction of healthy diploid cells are commonly present in the biopsies, the stoichiometry of CNVs might deviate from the expected stoichiometry of a fully homogeneous sample. As might be anticipated, we find no CNVs in the HB tissues (Figure 3A). Moreover, we find only 4 distinct CNVs in primary tumors, none of which we find in more than a single sample. In contrast, we found amplification of the q-arm of chromosome 1 in all three liver metastatic tumors. We also found recurrent (>1 patient) CNVs in chromosomes 8, 9, 11, 12, 13, and 22 in these metastatic tumors. In pleural effusion samples, CIN is most common, where we find recurrent (>1 patient) CNVs in every chromosome, with the exception of chromosome 14. Notably, while the malignant nature of all pleural effusions was pathologically confirmed, we did not detect CNVs in 2/10 pleural effusion samples. While 1q amplification was observed in every liver metastasis sample, pleural effusion samples showed this amplification in merely 3/10 samples. We find 8q amplifications in 6/10 pleural effusion biopsies. The q-arm of chromosome 8 harbors the prominent *MYC* oncogene which might explain why this region is amplified in the majority of samples. In agreement with prior work,^43^ CNVs appear to be mild in primary tumors^44^ and tend to become more abundant in metastatic^45^^,^^46^ and pleural effusion biopsies.

A translocation is the fusion of two different chromosomes. Because chromosomes occupy their own territory,^4^ seen as preferential intrachromosomal (“*cis*”) interactions, a fusion of two different chromosomes can be observed in Hi-C data as an extremely high number of interactions between chromosomes. Note that these would reveal themselves as interchromosomal (“*trans*”) interactions on the reference genome, but are in fact *cis* interactions. Breakpoints typically manifest themselves with a sharp “corner” where the putative breakpoint is found, and has previously been used to identify rearrangements.^21^^,^^47^^,^^48^ Translocations can occur as complex fusions between multiple chromosomes. For example, Figure 3B shows a rearrangement of chromosomes 1, 8, and 17 in the PB tumor of P04. Importantly, we find this rearrangement back in the metastatic tumor of the same patient, but additionally find several newly acquired breakpoints (arrows). We sought to quantify these more systematically by searching for sharp corners in the *trans* matrices followed by manual inspection and blinded classification (see STAR Methods for full details). In total, we found 222 putative breakpoints among all samples (Figure S2). Importantly, none of these breakpoints were found in healthy tissues. Among primary and metastatic samples, up to 14 breakpoints were detected in a single patient. In pleural effusion samples, this number increased to 33 (Figure 3C). Importantly, in all tumor biopsy types we find more patients with translocations than without, showing that translocations in breast cancer may occur more frequently than expected.

Overall, we find that SV becomes more excessive throughout cancer progression, both in terms of CNVs and translocations, with the most thoroughly rearranged genomes found in pleural effusion biopsies.

### Advanced cancer malignant pleural effusion samples show highly heterogeneous 3D genome features

**Figure 4:**
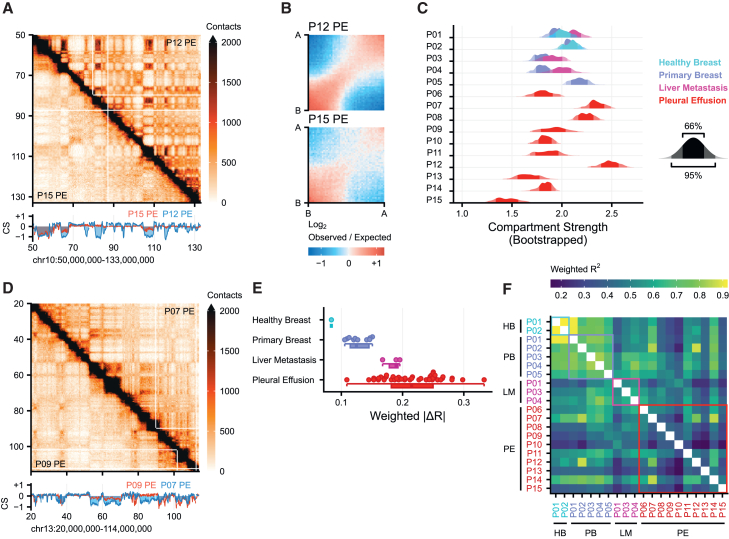
Compartmentalization diverges over progression (A) Example compartmentalization in two pleural effusion (PE) samples. Compartment score (CS) below indicates weak compartments in P15 whereas P12 has strong compartments. (B) Saddle plots showing interaction strength in a CS “quantile versus quantile” comparison. It indicates the strength of preferential A-A or B-B interactions relative to A-B interactions. Result represents averages over all chromosome arms. (C) Quantification of compartment strength for every sample, i.e., the strength of A-A and B-B interactions relative to A-B interactions. Displayed values represent kernel density estimates of 1000 bootstrap averages of compartment strength per chromosome arm. Darkest shading indicates 66th percentile interval (PI), medium shading indicates 95th PI. (D) Example compartmentalization in two different PE samples. CS indicates neutral-to-B compartment switches in both patients, but at different locations. (E) Boxplot of pairwise compartment dissimilarity, measured as weighted |ΔR|, within biopsy type. Higher values indicate more dissimilar compartments whereas lower values indicate more similar compartments. Box-and-whiskers plot indicate data distribution: boxes indicate the interquartile range (25%–75%), the whiskers extend to 1.5 time the interquartile range from the edge of the box or to the smallest or largest value. (F) Similarity of compartmentalization measured by weighted variance explained. Variance explained was calculated by correlating compartment scores per chromosome and subsequently weighting the squared correlation by chromosome length.

Our pleural effusion samples represent the most advanced and heavily pre-treated stage of breast cancer in our studies. Of note, due to the often long time interval between diagnoses and the frequent involvement of different hospitals throughout a patients’ treatment history, we were unable to acquire pleural effusion samples from patients of whom also primary or liver metastatic samples could be secured. Visual inspection of the Hi-C data from the pleural effusion samples revealed extensive heterogeneity at different levels of genome organization. When we take the q-arm of chromosome 10 as an example, we find that P12 and P15 have opposite manifestations of compartments: P12 has strong compartmentalization whereas P15 has limited compartmentalization (Figure 4A, compare also Figures 1F and 2D). The strength of compartmentalization can be summarized in a genome-wide fashion by calculating a “saddle-plot”, which displays the relative amounts of A-A, B-B, and A-B compartment interactions (Figure 4B). For P12, we observe stronger A-A and B-B interactions and weaker A-B interactions, reflecting a strong segregation of euchromatin and heterochromatin. In contrast, P15 still displays B-B interactions, but A-A interactions are barely visible. The degree of compartmentalization per chromosome arm can be quantified as the amount of A-A and B-B interactions versus A-B interactions, a metric known as the “compartment strength”.^49^ Using bootstrap averages over all arms, we quantified the compartment strength for every sample (Figure 4C). The liver metastatic biopsies have a slightly increased compartment strength compared to their paired primary tumors but the differences are minimal, especially when we consider the comparisons between patients. However, when we analyze the compartment strengths of pleural effusions, we find that they are characterized by a wide range of compartment strengths (Figure 4C). However, it is not only the compartment strength that is affected in pleural effusion samples, also compartment identity changes across the genome. For example, chromosome 13 shows stronger compartmentalization as observed in patient P07 compared to P09 (Figure 4D). When quantified genome-wide using a compartment dissimilarity metric, we find that pleural effusions have the greatest difference in compartment identity (Figure 4E). This result is compounded when we calculate the pairwise correlation of all our Hi-C samples (Figure 4F). Taken together, these data indicate that compartmentalization progressively becomes more heterogeneous with disease progression.

**Figure 5:**
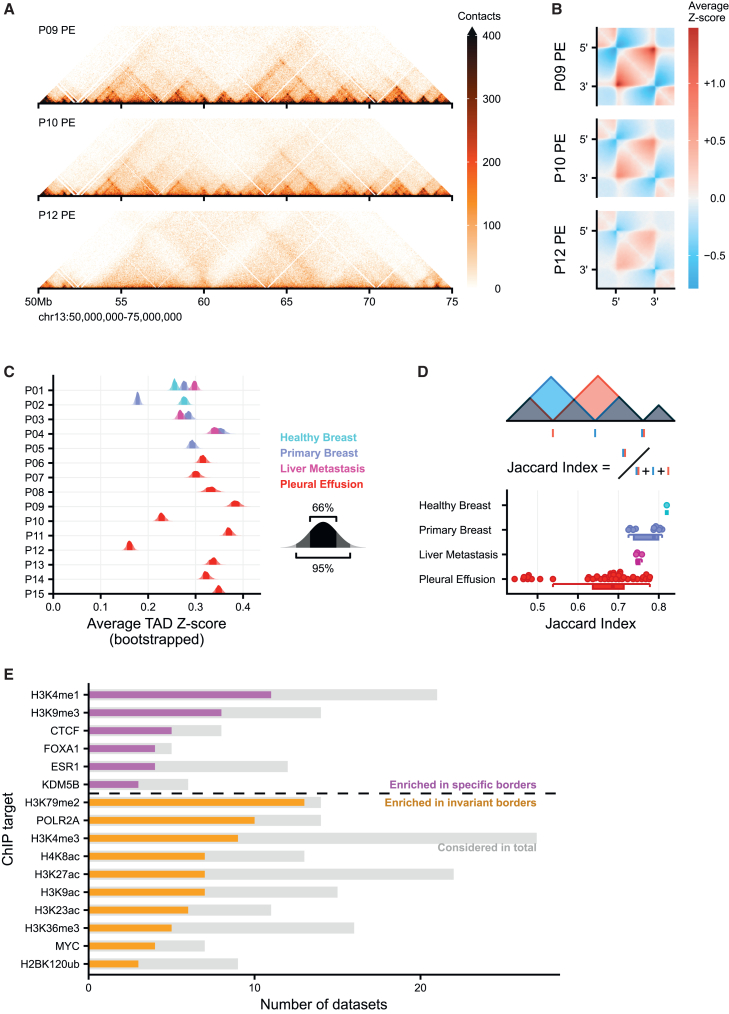
Rewiring of topologically associated domains (TADs) over progression (A) Example of TADs comparing three pleural effusion (PE) biopsies. (B) Aggregate TAD analysis (ATA) of the three biopsies presented in (A). Data shows averaged, *Z* score normalized TADs that have been rescaled to the same size. (C) Quantification of TAD strength in the ATA of (B). Data represent kernel density estimates of 1000 bootstrap averages resampling individual TADs per sample. (D) Boxplot of pairwise TAD border similarity as measured by the Jaccard index of border positions per biopsy type. Borders were considered overlapping when called within 60 kb of another border. Borders were called using insulation scores. Box-and-whiskers plot indicate data distribution: boxes indicate the interquartile range (25–75%), the whiskers extend to 1.5 time the interquartile range from the edge of the box or to the smallest or largest value. (E) Barplots show the significantly enriched mammary associated ChIPseq experiments in proximal to tumor-specific or tumor-invariant borders (see STAR Methods for details on calculation). Colored bars show the number of significant datasets, gray bars shows the total number of experiments considered in the analysis. Note that only chromatin marks with more than 3 significant experiments are plotted.

To understand whether the heterogeneity among pleural effusions at the level of compartmentalization is also observed at other levels, we zoomed in to the TAD level, where we observed various degrees of TAD strength for these samples (Figure 5A). For example, P09 showed clear TAD structures that were weaker in P10 and mostly absent in P12. To determine the average TAD strength, we identified TADs in all samples, performed rescaling and aggregated the Hi-C signal. We found that observed differences in TAD strength are a genome-wide phenomenon (Figure 5B). Across all samples, we found a broad range of TAD strengths (Figure 5C). However, we did not find a clear pattern in mean or variance of TAD strengths by comparing healthy tissue to primary tumor, primary tumor to metastasis or comparing pleural effusions to earlier biopsies. We interpret that TAD strength dysregulation might be commonplace, with both increases and decreases in intra-TAD interaction strength.

In addition to the TAD strength, the location of TAD boundaries may also differ between samples. While TADs were originally proposed to be stable across cell types,^7^ later studies showed that there may in fact be substantial variability in TAD boundary location depending on the cell type.^50^ Here, we consider the stability of TAD boundaries by computing the Jaccard index (JI): a set similarity metric that can be interpreted as a fraction of TAD boundaries that is shared between two samples. As expected, we found TAD boundaries to be stable between the two HB tissues (JI > 0.8). When we compare different tumor stages we find that with the progression of the disease the overlap in TAD boundary position decreases (Figure 5D). It is important to note that one pleural effusion biopsy (P12) is highly dissimilar to all other pleural effusion samples (average JI = 0.48). P12 also showed the lowest TAD strength (Figures 5A–5C), hinting at a defect in TAD establishment (see discussion). We performed an enrichment analysis (see STAR Methods for details) in which we took ChIP-seq experiments performed in mammary-related samples (healthy, tumor and cell lines) and determined whether the observed peaks were more associated with patient-specific or patient-invariant TAD boundaries (Figure 5E). We found that patient-invariant boundaries are significantly associated with chromatin features associated with promoters, such as H3K79me2, RNA Pol II, and H3K4me3. Patient-specific boundaries on the other hand were more associated with the poised enhancer mark H3K4me1, the heterochromatin mark H3K9me3 and CTCF. Together, these data support a model in which TAD strength and boundary position are affected by disease progression, however, there is not a single uniform response, resulting in highly dissimilar progressive metastatic lesions, with patient-distinct alterations in 3D genome architecture being enriched at distal regulatory enhancer elements, while promoter-involved contacts are more conserved between patients.

### ERα associates with multimegabase chromatin interactions

**Figure 6:**
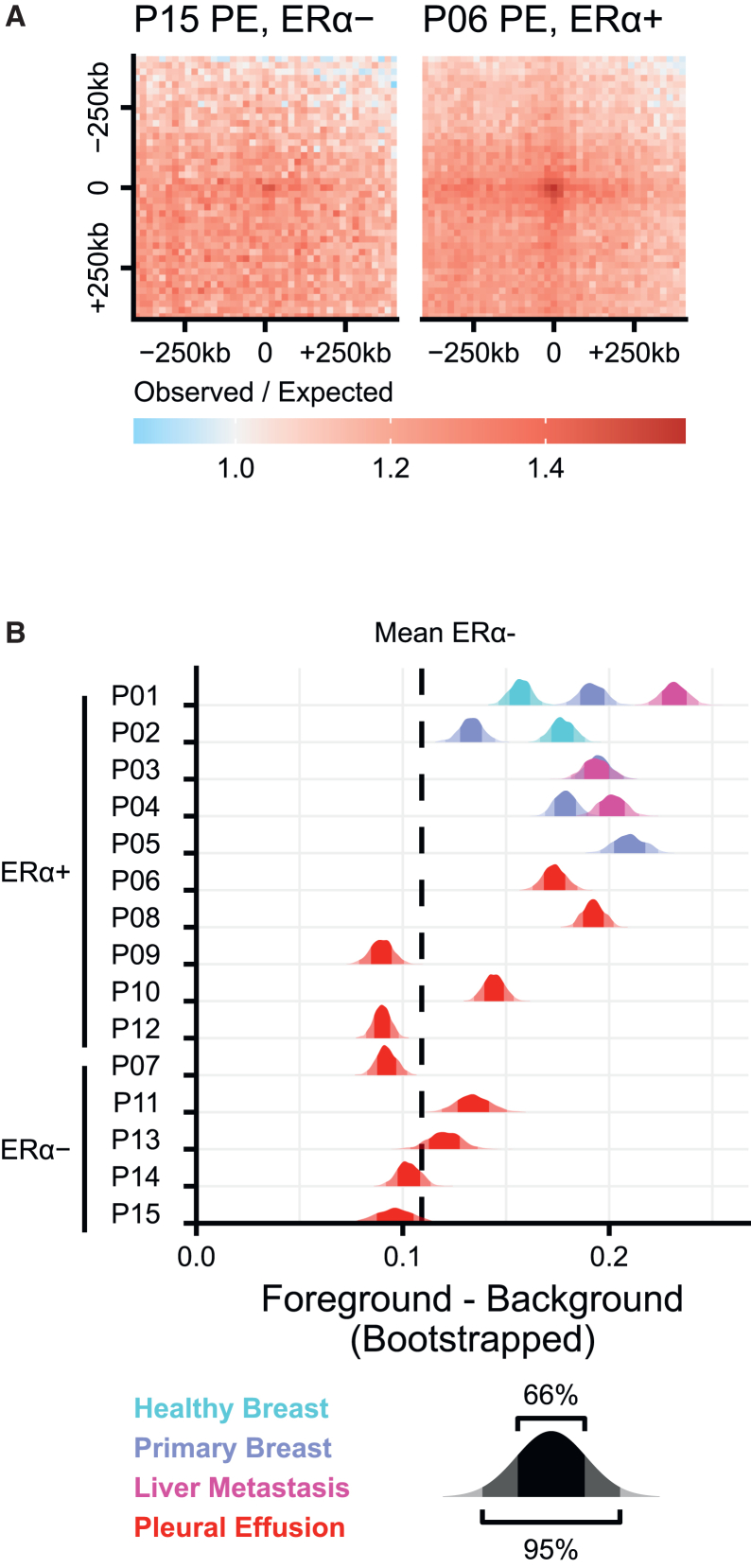
Distal ERα interaction frequency may be lost upon progression (A) Paired-end spatial chromatin analysis (PE-SCAn) showing distal (>3 Mb) interactions between clusters (≥3 peaks within 20 kb) of ERα binding as determined by ChIP-seq. Results of two pleural effusion biopsies with ERα- and ERα+ diagnoses are shown. (B) Quantification of PE-SCAns as described in (A) of all samples. Data shown are kernel density estimates of 1000 bootstraps of the average central 9 × 9 bins (foreground) minus the average of the 4 quadrants separated by these central bins.

ERα plays a crucial role in breast cancer development and progression and we next wanted to explore ERα chromatin binding features, in relation to 3D genome organization in our human breast cancer specimens. To investigate such interactions, we generated ERα ChIP-seq data for a subset of the pleural effusion samples. We identified high-density clusters of ERα binding sites as regions that have ≥3 ERα peaks with distances of less than 20 kb between subsequent peaks. We used paired-end spatial chromatin analysis (PE-SCAn) to search for distant interactions between clusters of ERα binding sites. Five of the ten pleural effusion samples are ERα– and, as expected, do not show long-range interactions between ERα binding clusters. On the other hand, in P06, which is a ERα+ pleural effusion sample, ERα binding clusters show preferential interaction (Figure 6A). These results show that ERα is associated with multimegabase chromatin interactions in cancer and that PE-SCAn can be used to measure this association.

We aimed to determine whether these interactions were also present in other samples in our Hi-C dataset. The HB, PB cancer and metastatic samples are all ERα+. Consistent with their ERα positive status of these samples we found that there were long-range interactions between linear clusters of ERα binding sites (Figure 6B). When we explored long-range interaction between ERα+ pleural effusion samples, we found that 3 out of 7 samples (i.e., P07, P09, and P12) showed a pattern of interactions more consistent with ERα-status. Interestingly, while the primary tumor of P07 stained ERα positive, the ERα staining on the pleural effusion samples, revealed these to be ERα− (Figure S3). Given that these interactions are prevalent in PB cancer and metastatic samples, it is likely that the absence of ERα-associated long-range interactions in these three pleural effusion samples has been acquired during disease progression.

## Discussion

In this study, we present *in situ* Hi-C data in breast cancer samples across four different stages of disease progression. While limited in size, our dataset has the unique feature of containing paired samples from primary tumors and metastatic lesions. Because we have included healthy tissues, we can also study how the 3D genome is affected during the transition from healthy to a diseased state. To our surprise, the changes in the 3D genome in tumor development were relatively limited. Both at the TAD level and at the level of compartmentalization, breast cancer samples did not drastically differ from healthy cells. The healthy samples were taken from tumor-adjacent normal tissue. We cannot formally exclude that the similarity in the 3D genome between healthy and tumor tissue is related to field cancerization.^51^ However, we deem this unlikely, because for patient 2 we find no SV in the healthy cells, whereas the tumor has accumulated multiple rearrangements, showing that the tumor and the surrounding tissue are genetically distinct. These data also indicate that despite the SV, the overall 3D genome was largely unaffected. A recent Hi-C analysis of three triple negative breast tumors^52^ found, similar to our data that compared to (unmatched) healthy tissue, contact frequency for all chromatin loops was largely unaffected. The authors also identified 227 loops specific for triple negative breast cancer. While these may be relevant for breast cancer progression, they only represent 1.6% of all loops in that dataset. We believe that this is consistent with our observation that there are no massive changes in the 3D genome when cells switch from a healthy to a diseased state.

In the transition from primary tumor to metastasis we observed substantial changes for one sample, however, for two other samples compartment identity is not drastically altered. Metastatic tumor cells have undergone extensive selection pressure to extravagate, survive in the bloodstream, grow out in the liver, and have survived extensive rounds of systemic therapeutics. Our results suggest that for a subset of tumors, 3D genome organization is maintained during this strong selection process. Analyses in a larger group of patients is required to appreciate whether these observations are exemplar for the general population, and whether deviations thereof may be associated with particular clinical features. Furthermore, whether these observations in the context of breast cancer can be translated to other cancer types, or generalized for the process of cancer progression as an overarching phenomenon in oncology, remains to be determined.

Systematic analysis of Hi-C maps of pleural effusion samples, which represent the most progressed breast cancer stage in our dataset, revealed that a large degree of heterogeneity of 3D genome features could be observed. These observations cover the identity and strength of compartments as well as in the position and strength of TADs. Given the relatively homogeneous 3D genome organization in primary and metastatic samples, it seems most likely that many changes in the 3D genome are acquired in this final step of tumor progression. It should be noted that the higher degree of plasticity at this tumor stage may also represent a form of survivorship bias, where tumor cells that have a higher plasticity are more likely to escape treatment pressure. It is a compelling idea that 3D genome plasticity may contribute to aggressiveness of the disease. This idea could be equally true for the high degree of genomic rearrangements that we observe in these samples. Unfortunately, our analyses do not allow us to distinguish cause and consequence of genetic variation introduced through genome instability and 3D genome variation that is introduced during disease progression.

In this light, the pleural effusion sample P12 is an interesting case. Our Hi-C analysis did not identify any gross genomic abnormalities (i.e., no copy number alterations, nor any structural variants). Interestingly, this sample has the weakest TAD score of all the samples in our dataset, but the strongest compartment score. It is well known that TAD formation, through cohesin-mediated loop extrusion, counteracts compartmentalization of the genome.^53^^,^^54^ The Hi-C profile of P12 is reminiscent of cells with a severely disrupted loop extrusion cycle, which would result in weakened TAD boundaries and increased compartmentalization. Mutations in cohesin subunits STAG2 have been associated with tumor formation in bladder cancer^55^ and Ewing’s sarcoma,^56^^,^^57^ among others. However, loss of the cohesin complex poses problems for proliferating cells, because it would result in the loss of sister chromatid cohesion and failure of cytokinesis.^58^ As the cells in P12 are still proliferating and bear no gross abnormalities, this would indicate that their cohesin complex can still faithfully hold sister chromatids together, but may have a strongly reduced loop extrusion capacity. Potential proteins that may explain this extrusion phenotype are NIPBL and MAU2. NIPBL, originally thought to be a loading factor, is now also recognized as a stimulator of loop extrusion.^59^^,^^60^^,^^61^ Furthermore, NIPBL forms a complex with MAU2^62^^,^^63^ and loss of MAU2 also results in a decrease of NIPBL protein levels.^54^ Loss or mutation of either of these proteins could lead to the observed phenotype of 3D genome organization. Whether the weakening of TADs confers additional plasticity for tumor cells to escape treatment pressure is an interesting question for the future.

Cohesin-mediated loop extrusion also results in one of the most noticeable features of a Hi-C map, i.e., (CTCF-anchored) chromatin loops.^16^^,^^64^ One of the limitations of the current study is that the sequencing depth does not allow for the calling of chromatin loops. This means that we were not able to extensively analyze any changes at this level of chromosome organization. It should be noted that particularly for the primary and metastatic tumor samples the amount of starting material is limited. This also means that deeper sequencing of existing Hi-C libraries will not necessarily yield an immediate increase in the resolution, because the complexity of the libraries is also limiting. It should also be noted that generating loop-resolution Hi-C matrices for large patient cohorts is prohibitively expensive at the moment. However, because sequencing costs have been consistently decreasing, we expect that in the future generating higher resolution datasets, using low input Hi-C methods^65^ will be a definite possibility. By using MNase digestion instead of restriction enzyme-based digestion (i.e., Micro-C^66^) it may also be possible to analyze promoter-enhancer interaction landscapes, although it is still an open question whether this protocol yields high-quality libraries from fresh frozen patient material.

Using breast cancer as a model, our study charts the 3D genome throughout the natural course of cancer development and progression, showing remarkably little alterations. In stark contrast, heavily pre-treated progressive malignant pleural effusions of metastatic breast cancer patients display highly divergent alterations in the 3D genome architecture between patients, reminiscent of the highly heterogeneous nature of progressive metastatic cancer.

### Limitations of the study

Procuring paired healthy, primary, and metastatic tumor samples is very challenging. Therefore the number of samples that we have been able to analyze in this study is limited. Also because the fresh frozen tumor material is more challenging to work with compared to fresh tissues or cell lines the complexity of our Hi-C maps is lower than what is typical. Although we are confident that at the level we have investigated the 3D genome in these samples the changes that we have observed are limited, future studies may use larger cohorts and more high-resolution methods such as Micro-C to be able measure changes at the level of promoter-enhancer interactions.

We were also unable to collect paired samples for metastatic lesions and pleural effusion samples. These samples would be very interesting to understand the evolution of the 3D genome for early metastasis to late stage metastasis, where the largest changes occur.

## Resource availability

### Lead contact

Further information and requests for resources and reagents should be directed to and will be fulfilled by the lead contact, Elzo de Wit (e.d.wit@nki.nl).

### Materials availability

This study did not generate unique reagents.

### Data and code availability

• Patient Hi-C and ERα ChIP-seq raw sequencing data (GRCh38/hg38 genome build) have been deposited at the European Genome-Phenome Archive (EGA), which is hosted by the EBI and the CRG, under accession number EGAS50000000444, while processed data are available at the Gene Expression Omnibus (GEO) database under accession number GSE273999.
• The original code generated for this work can be found at https://github.com/deWitLab/vdBrand_iScience (https://doi.org/10.5281/zenodo.15776141).
• Any additional information required to reanalyze the data reported in this paper is available from the lead contact upon request.

## Acknowledgments

We would like to acknowledge the Research High Performance Computing (RHPC) facility of the Netherlands Cancer Institute (NKI) to have enabled us to perform all the computations required to analyze the data generated, the NKI Genomics Core Facility for next-generation sequencing and bioinformatics support, and the NKI- AVL Core Facility Molecular Pathology & Biobanking (CFMPB) for supplying NKI-AVL Biobank material and lab support. We thank members of the de Wit and Zwart labs for valuable feedback, suggestions and input. In particular, we thank Dr. Joseph Siefert for performing analyses that did not end up in the paper and Mikhail Magnitov for assistance with the Hi-C mapping pipeline and loop calling. M. Donaldson Collier is supported by 10.13039/501100001711Swiss National Science Foundation (SNSF Post-doc Mobility). Work in the de Wit lab is supported by the 10.13039/501100003246Dutch Research Council (016.161.316, Vidi & VI.C.222.049, Vici) and the 10.13039/501100000781European Research Council (865459, “FuncDis3D”). Wilbert Zwart is supported by the Dutch Cancer Society and Alpe d’HuZes. This research was supported by Oncode Institute which is partly financed by the Dutch Cancer Society. Research at the Netherlands Cancer Institute is supported by institutional grants of the Dutch Cancer Society and of the Dutch Ministry of Health, Welfare and Sport.

## Author contributions

M.D.C., K.D.F., H.T., and S.G. generated data. T.v.d.B. performed data analysis. I.M.-P. and K.S. collected pleural effusion samples. Z.D. performed immunohistochemistry analysis. T.v.d.B., W.Z., and E.d.W. wrote the manuscript with input from all the authors.

## Declaration of interests

The authors declare no competing interests.

## STAR★Methods

### Key resources table

**Table:** 

REAGENT or RESOURCE	SOURCE	IDENTIFIER
**Antibodies**		

EpCAM (clone VU-1D9)	Invitrogen	Cat# MA1-10195; RRID: AB_11153547
ERα	Millipore	Cat# 06-935; RRID: AB_310305
ERα (immunohistochemstry) (clone SP1)	Roche Diagnostics	Cat# 790-4325; RRID: AB_2335977

**Biological samples**		

Human breast cancer specimens	NKI biobank	N/A

**Chemicals, peptides, and recombinant proteins**		

DAPI	Sigma-Aldrich	Cat# D9542
MboI	New England Biolabs	Cat# R0147L
Biotin-14-dATP	Life Technologies	Cat# 19524-016

**Critical commercial assays**		

NextSeq 500/550 High-Output v2.5 Kit (75 cycles)	Ilumina	Cat# 20024906

**Deposited data**		

Raw patient Hi-C and ERα ChIP-seq data for healthy breast, primary breast tumours, metastatic samples and pleural effusion samples	This study	EGA: EGAS50000000444
Processed patient Hi-C and ERα ChIP-seq data for healthy breast, primary breast tumours, metastatic samples and pleural effusion samples	This study	GEO: GSE273999

**Experimental models: Organisms/strains**		

Clinical parameters of the study subject can be found in Table S1	This study	Table S1

**Oligonucleotides**		

Hi-C adapters	Haarhuis et al.^54^	N/A

**Software and algorithms**		

SAMtools v1.12	Li et al.	RRID: SCR_002105
Picard v2.25.6	Broad Institute	RRID: SCR_006525
BEDTools v2.27.1	Quinlan and Hall	RRID: SCR_006646
deepTools v3.4.2	Ramírez et al.	RRID: SCR_016366
regioneR	Get et al.	https://bioconductor.org/packages/regioneR/
bioframe v0.3.3	Open2C	https://github.com/open2c/bioframe
distiller-nf	Open2C	https://github.com/open2c/distiller-nf
pairtools v0.3.0	Open2C	RRID: SCR_023038
cooler v0.8.11	Abdennur and Mirny^74^	RRID: SCR_024194
cooltools v0.5.1	Open2C	https://github.com/open2c/cooltools
GENOVA v1.0	Van der Weide et al.^75^	https://github.com/robinweide/GENOVA

### Experimental model and study participant details

All tissue used in this study was collected and stored in compliance with Dutch legislation, and pseudonymized by the NKI Data Protection Officer. This study was approved by the local medical ethics committee of the Netherlands Cancer Institute (Institutional Review Board (IRB) reference number: IRBdm20-007 and CFMPB411) and complies with the ethical principles of the Declaration of Helsinki. All patients provided informed consent for translational studies.

Demographic and clinocopathological details are provided in Table S1.

### Method details

#### Patient samples

All breast tissue samples were requested from the Netherlands Cancer Institute Biobank facility. Cryopreserved tissue samples were sectioned, 10×50μM sections were collected into a single tube and processed for Hi-C sequencing analysis. Malignant pleural effusions from patients with metastatic breast cancer were collected and processed immediately after drainage as described previously.^67^ In short, pleural fluid was centrifuged at 1600 g for 8 min, and erythrocytes were lysed, using the corresponding buffer (5 mM KHCO_3_, 75 mM NH_4_Cl, 400 μL 500 mM EDTA, up to 500 mL with ddH_2_O, pH: 7.4) for 10 min at room temperature. Cells were then either resuspended using 10% DMSO solution and stored at −80°C, or fixed with formalin and paraffin-embedded for future immunohistochemistry stainings. All tissue samples and malignant pleural effusion samples were scored by an expert pathologist for tumour cell percentage and Estrogen Receptor status. Only samples displaying a tumour percentage above 70% were processed for further analyses.

#### Immunohistochemistry

Immunohistochemistry of the FFPE tumor samples was performed on a BenchMark Ultra autostainer (Ventana Medical Systems). Briefly, paraffin sections were cut at 3 μm, heated at 75°C for 28 minutes and deparaffinized in the instrument with EZ prep solution (Ventana Medical Systems). Heat-induced antigen retrieval was carried out using Cell Conditioning 1 (CC1, Ventana Medical Systems) for 32 minutes at 950C. (EpCam) or 36 minutes at 950C. (ER).

ERα was detected using clone SP1 (Ready-to-Use, 32 minutes at 360C, Roche Diagnostics) and EpCam using clone VU-1D9 (1/400 dilution, 32 minutes at 370C, Invitrogen). Bound ERα antibody was detected using the UltraView Universal DAB Detection Kit (Ventana Medical Systems), while detection for EpCam was visualized using the OptiView DAB Detection Kit (Ventana Medical Systems). Slides were counterstained with Hematoxylin and Bluing Reagent (Ventana Medical Systems).

#### HE staining

Paraffin sections are cut at 2,5 um and dried at RT. HE staining is performed in the SAKURA Tissue-Tek Prisma® Plus Automated Slide Stainer. Slides are baked at 70°C for 20 minutes, followed by 2 times Xylene (Cat. # 24250502, BioSolve) incubation for 2 minutes to dewax. Slides are washed with 100% Ethanol and rehydrated in tap water followed by demineralized water. Sections are then incubated in Hematoxylin (Cat. # 4085-9005, VWR) for 1,5 minutes, after which they are washed with tap-water and Scott’s (Cat . # 3802901E, Leica Microsystems) and dehydrated with 100% ethanol before incubation of Eosine-y (Cat. # 4082-9002, VWR) solution for 1 minute. Slides are then washed with 100% ethanol to wash away excess Eosine, cleared in Xylene and cover slipped in the SAKURA Tissue-Tek Film Automated Coverslipper. A PANNORAMIC® 1000 scanner from 3DHISTECH was used to scan the slides at a 40x magnification.

#### ChIP-seq library preparation

Chromatin immunoprecipitation (ChIP) experiments were performed as described previously^68^ with the following modifications. Ten micrograms of anti-ERα (Millipore 06-935) antibody was prebound overnight to protein A Dynabeads magnetic beads (Invitrogen). The magnetic bead-chromatin complexes were harvested and washed 10 times with RIPA buffer [50 mmol/L HEPES (pH 7.6), 1 mmol/L EDTA, 0.7% Na deoxycholate, 1% NP-40, 0.5 M LiCl].

#### ChIP-seq analysis

ChIP-seq samples were processed using the *SPACCa* (v0.1.0) pipeline, publicly available at (https://github.com/sebastian-gregoricchio/SPACCa) using default parameters. Of note, FASTQ reads were mapped to the reference genome hg38/GRCh38, using the accelerated version of the Burrows-Wheeler Aligner (BWA-MEM2, v0.5.10).^69^ Reads were filtered based on mapping quality (MAPQ ≥ 20), and duplicated reads were removed. ChIP peaks over the input samples were called using MACS2^70^ filtering regions with q < 0.01.

#### Hi-C library preparation

For each sample, a minimum of 10×50μM sections (tissue samples) or ∼10×10^6^ of cryopreserved cells (pleural effusions) were cross-linked, digested using using MboI (New England Biolabs), and Hi-C library preparation was performed as previously described.^16^^,^^22^ Quality and quantification of the Hi-C libraries was assessed using the 2100 Bioanalyzer (Agilent, DNA 7500 kit). Biological replicates were pooled and subjected to sequencing using the Illumina NextSeq 550 (Illumina) in a 75bp paired-end setup. Only samples displaying a tumour percentage above 70% were further considered. An average of 100 million sequencing reads were obtained for each sample, enabling sufficient read coverage of the 3D genome.

#### Hi-C analysis

##### Data processing

Hi-C reads were mapped with Open2C *distiller-nextflow* pipeline v0.3.3.^71^ Reads were mapped with *bwa mem* v0.7.17-r1188^72^ to the GRCh38/hg38 human reference genome. Mapped reads were parsed and filtered with *pairtools* v0.3.0.^73^ Only reads with MAPQ>30 were considered for the Hi-C matrix generation. Cool files were created by *cooler* v0.8.11^7^^4^ and balanced via “balance” function with default parameters.

Most of the analysis on Hi-C data was performed using the GENOVA R package.^75^ Compartment scores were calculated at 100 kb resolution, signed with GC frequency and signing was synchronised between samples. Saddle plots were calculated at 50 quantile bins, from which compartment strength was calculated using the quantify function. Insulation scores were calculated at 20kb resolution and TADs were called from the insulation score using the min_strength = -Inf argument in the call_TAD_insulation function. ATA was performed in z-score normalised data at 20kb resolution. For calculating the Jaccard similarity between the boundaries, boundaries were first collapsed to unique boundaries by merging boundaries that were within 60kb of another boundary. PE-SCAns were calculated at 20kb resolution using clusters of ERα binding as input with the argument dist_thres = c(3e6, Inf) and quantified using size = 9, shape = “center_vs_quadrants”. Clusters of ERα binding were defined as having ≥ 3 ERα peaks with distances between subsequent peaks less than 20kb.

#### TAD boundary analysis

To analyse the overlap of TAD boundaries in pleural effusions with specific chromatin features we used pre-processed data from Cistrome.^76^ Data for human factors and histones were downloaded as batches from the Cistrome Data Browser website (http://cistrome.org/db/). Datasets were enriched for datasets from breast tissue or cancer by using the following inclusion and exclusion criteria. Inclusion criteria: (i) the cell line was described “MCF7”, “MCF-10A”, “MDA-MB-231”, “T47D” or “T47D-MTVL”, (ii) the cell type was described as “Epithelium”, “Mammary carcinoma cell” or “Human Breast Cancer Cell” (iii) the tissue was described as “Breast” or “Mammary Gland”. Exclusion criteria were (i) any tissues that were not described as “Breast”, “None” or “Mammary Gland” or (ii) have less than 1000 peaks. Moreover, for every factor/cell line combination, datasets were excluded that did not have the maximum ‘Peak Fold Change above 10’ metric.

To calculate overlap with TAD boundaries, TAD boundaries were expanded to a size of 60 kb and merged among experiments and filtered for non-disjoint ranges less than 100 kb in size. It was counted in how many PE samples these merged boundaries overlap with original, unmerged boundaries and split into separate sets based on the ubiquity. Sets that overlapped few samples were considered patient specific, whereas sets that overlapped many samples are considered patient invariant. For each combination of these sets and Cistrome datasets, it was counted whether a boundary overlapped a ChIPseq peak. From this, an 'observed + 1' over 'expected + 1' ratio was calculated, where the pseudocount of 1 was used to mitigate extreme ratios.

To find ChIP-seq datasets that were patterned with regards to how ubiquitous TAD boundaries are, we used a likelihood test. For the test we fitted two models with the observed/expected ratio as the dependent variable. The simpler model was a 0th degree polynomial (independent of boundary ubiquity), whereas the more complex model was a 1st degree polynomial with boundary ubiquity as predictor.

#### Copy number estimates

The genome was tiled at 1Mb resolution. For every tile, the following metrics were calculated: (1) the number of 5′ read ends from valid, unduplicated Hi-C read pairs that mapped to the tile (2) the number of MboI restriction sites in the tile (3) average GC-frequency and (4) average mappability. The locfit function from the homonymous R package^77^ with the argument family = “poisson”, was used to regress out the influence of mappability, GC frequency and restriction sites from the read counts to obtain normalised counts. The log_2_ ratio of the normalised counts over the genome-wide median of counts was used as input for segmentation analysis using the DNAcopy R/Bioconductor package.^39^

#### Translocation calling

Translocations were called on unbalanced data by performing normalisation steps followed by a 2D convolution filter geared towards detecting corners. Unbalanced data at 500 kb resolution was filtered for 1D bins that had more than 2000 reads assigned to them, and read pairs mapped to cis chromosomes were set to 0.

To normalise for copy number variation (CNV), a negative binomial generalised linear model, using glm.nb from the MASS R package,^78^ was used to fit the read count per cell as a function of the product of the log_10_ transformed row- and column-means for a random subset of 10^5^ cells. This model was then used to predict an ‘expected’ value for every cell. The CNV normalised data was then computed as follows:

$$\log_{2}\frac{\text{observed}+\varphi }{\text{expected}+\varphi }$$

Wherein indicates a pseudo-count that was set to 20.

Next, the data was normalised for compartmentalisation. An eigendecomposition was computed using the eigs_sym from the RSpectra R package^79^ with the which argument set to “LA”. The crossproduct of the first eigenvector was used to model the CNV-normalised data using the rlm function from the MASS package without intercept.^78^ The residuals of this model were taken as normalised data.

Per chromosome pair, the Hi-C matrix was treated as an image and passed through a 3x3 median filter. The resulting image was subjected to a 2D convolution with a 7x7 corner filter using the filter2 function in the EBImage R/Bioconductor package^80^ with the boundary argument set to “replicate”. The intensity was floored at 1 to eliminate anti-pattern values and the results of 4 different orientations of the corner filter were summed to produce a new image representing how well an original pixel resembles a corner. This new image was binarised at an intensity of 100 and the areas were dilated with a 21x21 square brush to merge adjacent areas. The pixel with the highest original Hi-C read counts in each disconnected area served as a candidate breakpoint.

A series of plots of 60Mb around candidate breakpoints at 200kb resolution was saved and manually classified. The classifier was blinded to patient identity, and plots were displayed in randomised order during classification. The following qualitative criterions were used (1) intensity of breakpoint should visually stand out above background (2) if multiple potential breakpoints are in the same view, the considered breakpoint is the most intense one and (3) in noisy regions, such as telomeres, centromeres or otherwise regions with low mappability, evidence of the break must propagate outside the problematic region. Please note that the number of translocations is relatively small and is therefore unlikely to affect the 3D genome analyses.

### Quantification and statistical analysis

ChIP-seq datasets that were associated with tumor specific and tumor invariant TAD boundaries identified using a likelihood-ratio test. The false discovery rate threshold was 0.001. Since no additional statistical analyses were performed, no related figures or tables were included in the manuscript.

## Supplemental information

Document S1. Figures S1–S3

Table S1. Overview of the demographic and clinicopathological details of the study subjects, related to Figure 1
