# Supplementary material for: A 3D genome compendium of breast cancer progression
Source: iScience. 2025 Aug 5;28(9):113268. doi: 10.1016/j.isci.2025.113268 (PMC12391274; doi:10.1016/j.isci.2025.113268)
Supplement: Document S1. Figures S1–S3 [file mmc1.pdf]

iScience, Volume *28*

Supplemental information

A 3D genome compendium of breast cancer progression

Teun van den Brand, Maria Donaldson Collier, Koen D. Flach, Sebastian Gregoricchio, Isabel Mayayo-Peralta, Zhanna Dauyey, Karianne Schuurman, Hans Teunissen, Wilbert Zwart, and Elzo de Wit

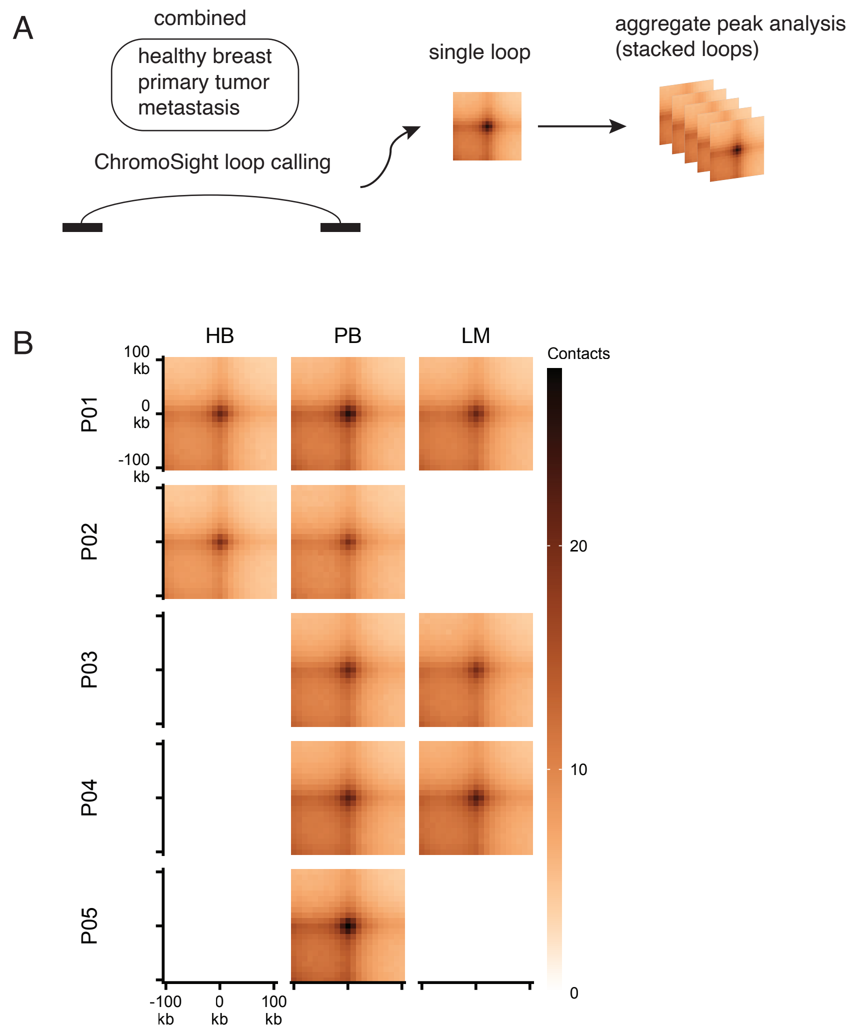

Figure S1: Aggregate peak analysis on healthy breast, primary tumors and metastatic samples.

A) Explanation of the APA analysis. Loops have been called on the collective Hi-C maps. By averaging the signal of all the loops an average loop signal is calculated.

B) Results from the individual Hi-C experiments in healthy breast (HB), primary breast cancer (PB) and liver metastasis (LM).

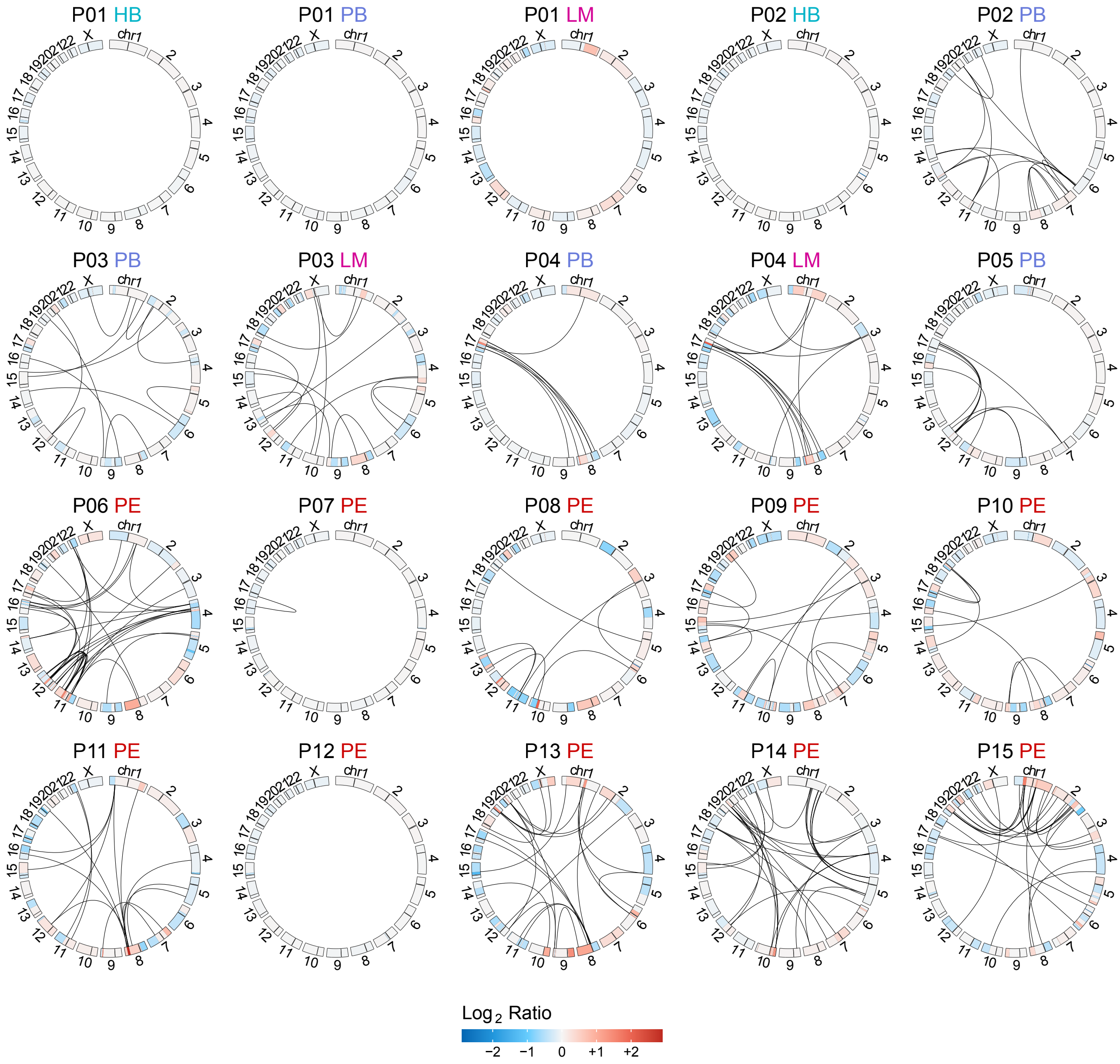

Figure S2: Overview of structural variation.
Plots showing chromosomes layed-out in a circle and coloured by copy number estimates as measured by the segmented $\log_2$ ratio of normalised reads to grand median per bin. Intrachromosomal rearrangements found by manual classification are indicated by lines between chromosomes.

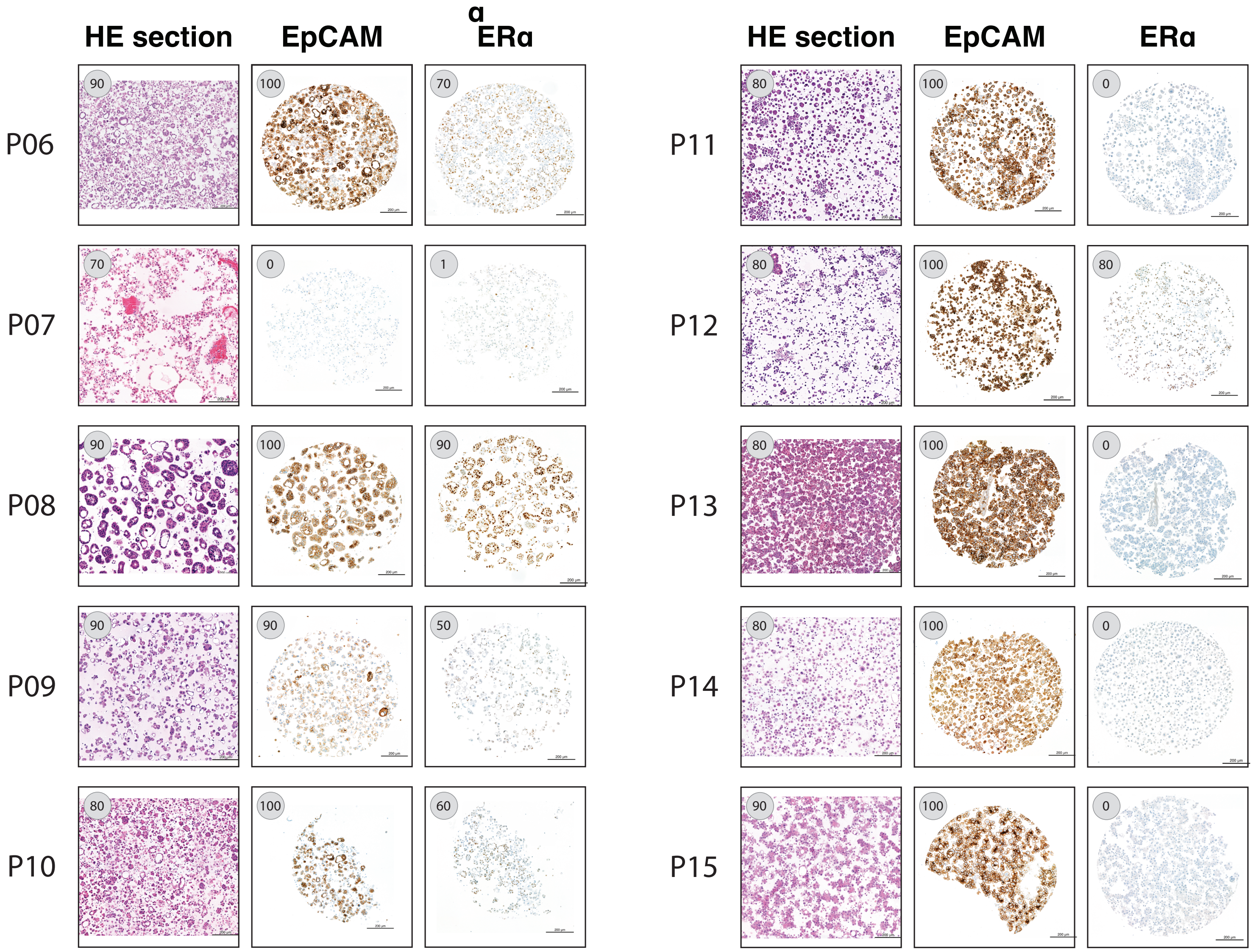

Figure S3: Tumour cell percentage and ERα staining for pleural effusions samples. Hematoxylin and eosin stain images and immunohistochemistry (IHC) analysis for EpCAM and ERα shown for the pleural effusion tumor samples. Grey circles indicate tumour cell percentage for the H&E stain, EpCAM positive cells for the EpCAM IHC and ERα positive cells for the ERα IHC.
